# Evolutionary dynamics of plastomes in coscinodiscophycean diatoms revealed by comparative genomics

**DOI:** 10.3389/fmicb.2023.1203780

**Published:** 2023-06-15

**Authors:** Feng Liu, Yichao Wang, Hailong Huang, Nansheng Chen

**Affiliations:** ^1^CAS Key Laboratory of Marine Ecology and Environmental Sciences, Institute of Oceanology, Chinese Academy of Sciences, Qingdao, Shandong, China; ^2^Marine Ecology and Environmental Science Laboratory, Laoshan Laboratory, Qingdao, Shandong, China; ^3^Center for Ocean Mega-Science, Chinese Academy of Sciences, Qingdao, Shandong, China; ^4^Chinese Academy of Fishery Sciences, Beijing, China; ^5^School of Marine Sciences, Ningbo University, Ningbo, Zhejiang, China

**Keywords:** diatom, plastome, Coscinodiscophyceae, gene duplication, inverted repeats, phylogenomic analysis

## Abstract

To understand the evolution of coscinodiscophycean diatoms, plastome sequences of six coscinodiscophycean diatom species were constructed and analyzed in this study, doubling the number of constructed plastome sequences in Coscinodiscophyceae (radial centrics). The platome sizes varied substantially in Coscinodiscophyceae, ranging from 119.1 kb of *Actinocyclus subtilis* to 135.8 kb of *Stephanopyxis turris*. Plastomes in Paraliales and Stephanopyxales tended to be larger than those in Rhizosoleniales and Coscinodiacales, which were due to the expansion of the inverted repeats (IRs) and to the marked increase of the large single copy (LSC). Phylogenomic analysis indicated that *Paralia* and *Stephanopyxis* clustered tightly to form the Paraliales-Stephanopyxales complex, which was sister to the Rhizosoleniales-Coscinodiscales complex. The divergence time between Paraliales and Stephanopyxales was estimated at 85 MYA in the middle Upper Cretaceous, indicating that Paraliales and Stephanopyxales appeared later than Coscinodiacales and Rhizosoleniales according to their phylogenetic relationships. Frequent losses of housekeeping protein-coding genes (PCGs) were observed in these coscinodiscophycean plastomes, indicating that diatom plastomes showed an ongoing reduction in gene content during evolution. Two *acpP* genes (*acpP1* and *acpP2*) detected in diatom plastomes were found to be originated from an early gene duplication event occurred in the common progenitor after diatom emergence, rather than multiple independent gene duplications occurring in different lineages of diatoms. The IRs in *Stephanopyxis turris* and *Rhizosolenia fallax*-*imbricata* exhibited a similar trend of large expansion to the small single copy (SSC) and slightly small contraction from the LSC, which eventually led to the conspicuous increase in IR size. Gene order was highly conserved in Coscinodiacales, while multiple rearrangements were observed in Rhizosoleniales and between Paraliales and Stephanopyxales. Our results greatly expanded the phylogenetic breadth in Coscinodiscophyceae and gained novel insights into the evolution of plastomes in diatoms.

## Introduction

Diatoms are one of the most successful phytoplankton groups in contemporary oceans, accounting for roughly 20% of the primary productivity on Earth (Falkowski et al., 1998), as well as being the primary biological mediators of the silica cycle in oceans (Sumper and Brunner, 2008). Some diatom species are widely used as initial feeding in aquaculture, while others have demonstrated great potential to be important metabolites in the fields of biomedicine, bioenergy and biomaterials (e.g., Hemaiswarya et al., 2011; Kiatmetha et al., 2011; Nurachman et al., 2012). Some diatoms species proliferate rapidly and massively in regional waters, resulting in the development of harmful algal blooms (HABs) which have serious negative impact on economy and the ecological environment (Armbrust, 2009).

Diatoms are an extraordinarily diverse lineage in evolution and morphology, and it is believed that there are as many as 100,000 extant species around the world (Mann and Droop, 1996; Mann et al., 2021). Up to now, more than 18,200 species of diatoms have been effectively described in taxonomy around the world (Guiry and Guiry, 2023). There is no doubt that a large number of cryptic species have not yet been properly resolved due to their morphological similarities. Current classification system of diatoms based on molecular biological data, combined with morphological features, sexual reproduction, and fossil evidence (Medlin and Kaczmarska, 2004; Medlin and Desdevises, 2020), has revealed that the Bacillariophyta phylum could be divided into two subphyla, Bacillariophytina and Coscinodiscophytina. Bacillariophytina harbors two classes, Bacillariophyceae (pennates) and Mediophyceae (polar centrics), harboring 78.7 and 10.2% of diatom species, respectively. Coscinodiscophytina contains a single class Coscinodiscophyceae (radial centrics), which thus far has 1,548 species, occupying 8.5% (Yu et al., 2018; Guiry and Guiry, 2023).

Diatoms are unicellular photosynthetic heterokont algae that are globally distributed in marine and freshwater environments. Diatoms represent a lineage of photosynthetic heterokonts which acquired their chloroplasts from a red algal ancestor by secondary endosymbiosis that took place around one billion years ago (Bhattacharya et al., 2007), thus their chloroplasts are surrounded by four layers of membranes. Diatom nuclear genomes sequenced thus far harbored genes from a heterotrophic host cell, bacterial donors, and red algal endosymbionts, displaying mosaic nature of their genetic material (Armbrust et al., 2004; Bowler et al., 2008).

Nevertheless, in the latest 10 years, the number of diatom chloroplast genomes (plastid genomes, plastomes) increased rather rapidly driven by high throughput DNA sequencing technologies. As of 1st May 2023, more than 150 diatom plastomes from at least 130 species have been deposited in the GenBank database. The sequenced diatom plastomes display a canonical quadripartite organization with two inverted repeats (IRs) between a large single copy (LSC) region and a small single copy (SSC) region, and harbor a core set of 150 - 165 canonical genes (e.g., Kowallik et al., 1995; Oudot-Le Secq et al., 2007; Hamsher et al., 2019). Diatom plastomes show a reduced gene content in comparison with red algal plastomes (230–254), indicating that many chloroplast genes have been lost or transferred to the nuclear genome after secondary endosymbiosis (Lommer et al., 2010; Lang and Nedelcu, 2012). Meanwhile, some genes were acquired via lateral gene transfer from different sources (e.g., plasmids, bacteria), leading to the expansion of plastome sizes (Brembu et al., 2014; Ruck et al., 2014). Although they show a high degree of similarity in genome architecture and core gene set (Sabir et al., 2014; Ruck et al., 2017; Wang et al., 2022), diatom plastomes have undergone tremendous alterations in genome size, IR size and gene content, and gene order through evolutionary history of diatoms (Yu et al., 2018; Gastineau et al., 2021). Plastomes of some closely related intrageneric species or even intra-order species displayed highly conserved gene order and genome structure (Sabir et al., 2014; Hamsher et al., 2019; Xu et al., 2021; Zhang and Chen, 2022).

Thus far, only six plastomes from four genera in two orders (Rhizosoleniales and Coscinodiacales) in coscinodiscophycean diatoms have been sequenced (Table 1). Considering the rich morphological diversity of coscinodiscophycean diatom species, the important evolutionary status of this class which represents a basal lineage in the evolution of early diatoms, and their global distribution and important ecological significance, it is very necessary to fill this gap to understand their evolution trend and relationships. This study focused on the evolution of plastomes in coscinodiscophycean diatoms. Plastomes from six coscinodiscophycean diatom species including *Guinardia delicatula*, *Guinardia striata*, *Actinocyclus* sp., *Coscinodiscus granii*, *Stephanopyxis turris*, and *Paralia sulcate*, which were collected in the Jiaozhou Bay of China, were constructed in this study and compared with plastome sequences deposited in the GenBank database to ascertain their evolutionary dynamics.

**TABLE 1:** General features of 12 diatom plastomes in Coscinodiscophyceae for comparative analysis.

Species	Order	Accession number	Size (bp)	G + C (%)	LSC (bp)	SSC (bp)	IR (bp)	Canonical genes**	References
								**PCGs/rRNAs/tRNAs/sRNAs**	
*Guinardia delicatula*	Rhizosoleniales	OM827252	123,772	32.39	61,109	39,097	11,783	130/3/27/2	This study
*Guinardia striata*	Rhizosoleniales	OM827251	121,778	31.68	59,939	38,657	11,591	130/3/27/2	This study
*Guinardia striata*	Rhizosoleniales	MG755796	122,145	32.26	59,710	38,869	11,783*	129/3/27/2	Yu et al., 2018
*Rhizosolenia setigera*	Rhizosoleniales	MG755793	121,011	32.17	58,541	38,332	12,069	129/3/27/2	Yu et al., 2018
*Rhizosolenia fallax*	Rhizosoleniales	MG755802	125,283	30.20	59,165	28,184	18,967	122/3/27/2	Yu et al., 2018
*Rhizosolenia imbricata*	Rhizosoleniales	KJ958482	120,956	31.76	61,244	27,482	16,115	123/3/27/2	Sabir et al., 2014
*Actinocyclus* sp.	Coscinodiacales	OM827248	120,465	31.04	59,224	38,731	11,255	129/3/27/2	This study
*Actinocyclus subtilis*	Coscinodiacales	MG755799	119,120	29.42	59,040	38,042	11,019	130/3/27/2	Yu et al., 2018
*Coscinodiscus granii*	Coscinodiacales	MW561225	123,615	31.18	60,117	37,498	13,000	131/3/27/2	This study
*Coscinodiscus radiatus*	Coscinodiacales	KC509521	122,213	30.41	60,402	36,643	12,584	131/3/27/2	Ruck et al., 2014
*Paralia sulcata*	Paraliales	OM827250	132,157	30.97	68,214	36,673	13,635	128/3/27/2	This study
*Stephanopyxis turris*	Stephanopyxales	OM827249	135,791	30.37	68,199	27,372	20,110	128/3/27/2	This study

*One of IR gene clusters was inverted in the *Guinardia striata* plastome (MG755796), which resulted in two IR gene clusters being arranged in a forward direction instead of a reverse direction.

**Multicopy genes were counted once, e.g., the canonical genes located in IRs as well as the tRNA with duplication (Supplementary Table 3).

## Materials and methods

### Sampling and isolation of diatoms

Water samples were collected in Jiaozhou Bay (36°00′–36°12′N, 120°10′–120°24′E) of China from August 2020 to December 2021 onboard the R/V Chuangxin, which was operated by the Jiaozhou Bay National Marine Ecosystem Research Station. Single cells of each diatom species were isolated using single-cell capillary methods from water samples. Six unialgal diatom strains (CNS00558, CNS00513, CNS00114, CNS00554, CNS00378, and CNS00428) were successfully cultured in L1 medium with 1 ‰ volume fraction Na_2_SiO_3_. The culture was maintained at 18–20°C, 2,000–3,000 Lux in the photoperiod of 12 h light-12 h dark.

### DNA extraction, and sequencing

Total genomic DNA for each diatom strain was extracted using the DNAsecure Plant Kit (Tiangen Biotech, Beijing, China). After purification, genomic DNA samples were fragmented into a size of 350 bp using Covaris S220 ultrasonic crater (Covaris, USA) for library construction. The DNA libraries were sequenced using a NovaSeq 6000 platform (Illumina, San Diego, CA, USA), yielding about 5-Gb sequencing data of paired-end reads with 150 bp in length. Illumina sequencing raw data were trimmed using Trimmomatic v0.39 with the parameters: LEADING:3 TRAILING:3 SLIDING WINDOW:4:15 MINLEN:75 (Bolger et al., 2014).

### Identification of coscinodiscophycean diatom species

**FIGURE 1:**
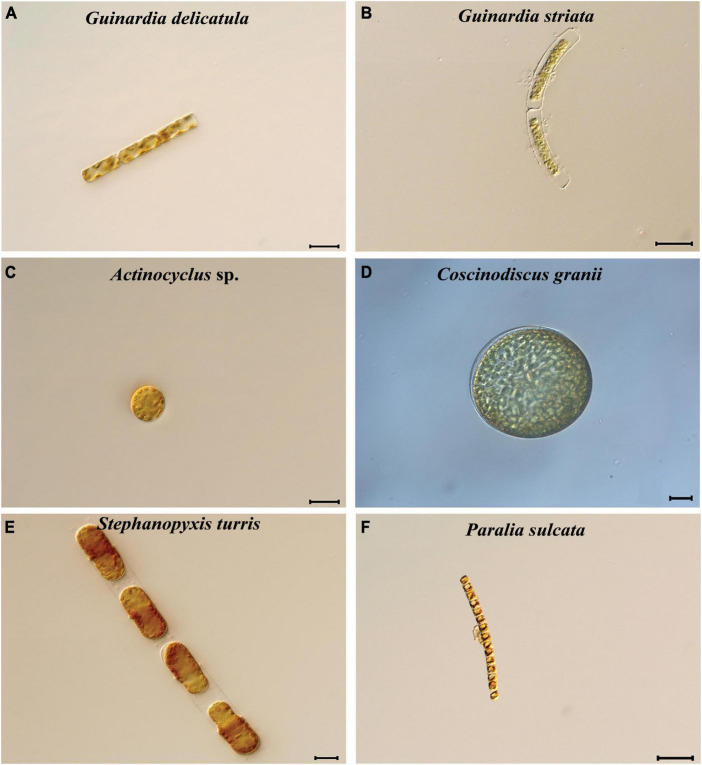
Micrographs of six diatom species in class Coscinodiscophyceae. **(A)** *Guinardia delicatula*. **(B)** *Guinardia striata*. **(C)** *Actinocyclus* sp. **(D)** *Coscinodiscus granii*. **(E)** *Stephanopyxis turris*. **(F)** *Paralia sulcate*. Bar = 20 μm.

Species identification of six coscinodiscophycean diatoms was performed based on comparative analysis of their full-length 18S rDNA sequences as well as their morphological characteristics (Figure 1). Full-length 18S rDNA sequences of these diatom strains were assembled using Illumina sequencing results using the GetOrganelle v1.7.4.1 (Jin et al., 2020) and SPAdes v3.14.0 (Bankevich et al., 2012). The 18S rDNA sequences of coscinodiscophycean diatoms downloaded from the GenBank database were used as reference sequences (Supplementary Table 1). Multiple sequence alignments of 18S rDNA sequences were conducted by using ClustalX v1.83 with the default settings (Thompson et al., 1997). Evolutionary relationships were evaluated based on the analysis of the similarity of the 18S rDNA sequences using MEGA v7.0 (Kumar et al., 2016). 18S rDNA sequences of strains CNS00558, CNS00513, CNS00554, CNS00378, and CNS00428 displayed very high similarities (>99.6%) with the reference sequences deposited in the GenBank database, respectively (Supplementary Table 2). According to molecular data and their morphological characteristics, these five strains were identified as *Guinardia delicatula*, *Guinardia striata*, *Coscinodiscus granii*, *Stephanopyxis turris*, and *Paralia sulcate*, respectively. Strain CNS00114 shared the highest similarity with *Actinocyclus* sp. (X85401), reaching 98.86% (Supplementary Table 2), suggesting that this strain represents an undescribed species in *Actinocyclus*.

### Construction and annotation of plastomes

Clean reads were used to assemble complete plastome sequences using GetOrganelle v1.7.4.1 (Jin et al., 2020). To verify the completeness of these plastomes, GetOrganelle generally exported consistent assembly results with the same raw reads using different parameters, when a complete plastome was obtained (Jin et al., 2020). Whether an assembled plastome was circular was further confirmed by aligning short reads against the assembled plastome sequence using the MEM algorithm of BWA v0.7.17 (Li and Durbin, 2009) and SAMtools v1.10 (Li et al., 2009). Circular plastome was supported by perfect alignments of short reads along the entire length of assembled plastome sequence. The alignment was visualized using IGV v2.7.2 (Thorvaldsdottir et al., 2013). Annotation was conducted using MFannot^1^ and NCBI’s ORF Finder,^2^ which was further improved using NCBI’s Sequin v15.10. For best accuracy of comparative analysis, we had checked and re-annotated coscinodiscophycean diatom plastomes deposited in the GenBank database.

### Phylogenomic and synteny analysis

Due to the absence of four protein-coding genes (PCGs) (*bas1*, *ycf88*, *ycf89*, and *ycf90*) in *Triparma laevis* (Bolidophyceae, Ochrophyta) plastome when compared with coscinodiscophycean plastomes, a total of 116 PCGs from 12 coscinodiscophycean plastomes as well as *Triparma laevis* plastome (Tajima et al., 2016) were extracted and concatenated for phylogenomic analysis. The amino acid (AA) sequences of each PCG were individually aligned and checked using MAFFT v7.471 (Katoh and Standley, 2013), and ambiguously aligned regions were trimmed by trimAl v1.4 (Capella-Gutierrez et al., 2009). These AA datasets were concatenated using PhyloSuite v1.2.2 (Zhang et al., 2020). The best-fit model was tested and identified using ModelFinder (Kalyaanamoorthy et al., 2017). Phylogenomic tree was constructed by IQ-TREE v1.6.12 (Trifinopoulos et al., 2016) using default parameters with 5,000 ultrafast bootstrap analysis (Minh et al., 2013). *T. laevis* was used as the outgroup. The AA sequences of AcpP (AcpP1 and AcpP2) were aligned by ClustalX v1.83 with default settings (Thompson et al., 1997). The phylogenetic relationships were inferred with the Maximum Likelihood (ML) method based on the JTT matrix-based model (Jones et al., 1992) using MEGA v7.0 (Kumar et al., 2016). There were 113 positions in the final dataset of AcpP. Synteny analysis of plastomes was performed using the progressiveMauve software of the package Mauve v2.3.1 (Darling et al., 2010). Comparative illustration of coscinodiscophycean plastomes was conducted using circos v0.69 (Krzywinski et al., 2009).

### Divergence time estimation

Phylogenetic relationship and molecular dating were analyzed by calculating the codon evolution rate of nucleotide (nt) sequences of 109 PCGs which were shared by plastomes of selected diatom species and *Ectocarpus siliculosus* (Phaeophyceae, Ochrophyta). *E. siliculosus* was used as outgroup with its known fossil time. The nt sequences were aligned using MAFFT v7.471 and concatenated using PhyloSuite v1.2.2 (Zhang et al., 2020). The phylogenetic tree was constructed using IQ-TREE v1.6.12 (Trifinopoulos et al., 2016), and molecular dating was conducted using the PAML package v4.8a (Yang, 2007). Estimation of substitution rate was performed using baseml, and estimation of divergence times with the approximate likelihood method was carried out using mcmctree. The phylogenetic tree was displayed in Figtree v1.4.3 and visualized with 95% highest posterior density interval (HPD) for each node. Three calibrations of internal nodes, including *E. siliculosus* at 176–202 MYA (Matari and Blair, 2014), *R. setigera* at 90–93 MYA (Sinninghe-Damsté et al., 2004), and Thalassiosirales at 40–50 MYA (Sims et al., 2006), were conducted in divergence time estimation.

## Results and discussion

### Molecular features of coscinodiscophycean plastomes

Complete plastome sequences of six coscinodiscophycean diatom species which exhibited rich diversity in cell morphology and cell size (Figure 1) were successfully assembled. These plastomes ranged in size from 120.5 kb in *A. curvatulus* to 135.8 kb in *S. turris*, and their G + C content was from 30.37% in *S. turris* to 32.39% in *G. delicatula* (Table 1), which were in the range of reported diatom plastomes (Ruck et al., 2017; Liu et al., 2021a,b; He et al., 2022; Wang et al., 2022). Similar to the reported diatom plastomes (e.g., Kowallik et al., 1995; Oudot-Le Secq et al., 2007; Tanaka et al., 2011; Hamsher et al., 2019), these newly assembled plastomes were mapped as canonical circular quadripartite structures with two IRs separating LSC and SSC. The conserved *psbD-psbC* overlapping region was detected in these coscinodiscophycean plastomes, but also in plastomes of Bacillariophyta and Ochrophyta (Kowallik et al., 1995; Liu et al., 2017). However, comparative analysis reveals that the *psbD-psbC* overlapping regions in the *Stephanopyxis* and *Paralia* plastomes reduced from the original 53 to 17 bp (GTGGAAACGCCCTTTAA), due to a single base mutation (T→C) which should have occurred in the common progenitor of *Stephanopyxis* and *Paralia*, and *psbC* started with GTG instead of ATG.

Combined the other six plastomes of coscinodiscophycean diatom species deposited in the GenBank database, we found that these 12 plastomes represented four orders in the class Coscinodiscophyceae (Table 1), which enabled us to obtain valuable evolutionary clues. The 135.8-kb *Stephanopyxis* plastome was the largest one found in Coscinodiscophyceae thus far, followed by the 132.2-kb *Paralia* plastome, and then the plastomes in Rhizosoleniales and Coscinodiacales. There is no significant difference in plastome sizes between Rhizosoleniales and Coscinodiacales (Table 1). Plastomes in Paraliales and Stephanopyxales tended to be larger than those in Rhizosoleniales and Coscinodiacales (Ruck et al., 2014; Sabir et al., 2014; Yu et al., 2018), which was caused not only by the expansion of the IR but also by the marked increase of the LSC. The size of LSC in Paraliales and Stephanopyxales is at least 8.0 kb larger than that in Rhizosoleniales and Coscinodiacales (Table 1). Numerous expanded intergenic regions were caused by accepting foreign DNA fragments in the *Stephanopyxis* and *Paralia* plastomes, which made them less compact when compared with plastomes in Rhizosoleniales and Coscinodiacales. However, it is difficult to trace the exact origin of some foreign fragments detected in diatom plastomes thus far, although a small fraction has been identified to be from diatom plasmids (Brembu et al., 2014; Ruck et al., 2014).

### Divergence time estimation of coscinodiscophycean plastomes

**FIGURE 2:**
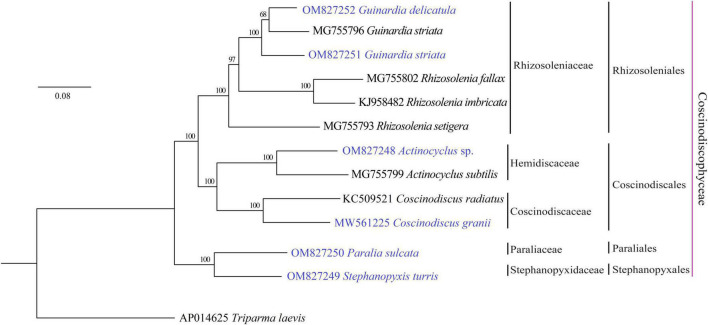
Maximum Likelihood (ML) phylogenomic tree of coscinodiscophycean diatom species based on concatenated amino acid sequences of 116 PCGs shared by plastomes of these diatoms as well as *Triparma laevis* as outgroup. Numbers at the branches represented bootstrap values. Branch lengths were proportional to the amount of sequence change, which were indicated by the scale bar below the trees.

Phylogenomic analysis based on AA sequences of 116 PCGs shared by the 12 coscinodiscophycean plastomes as well as the *T. laevis* plastome (Tajima et al., 2016) as the outgroup revealed that the sampled coscinodiscophycean diatom species were grouped into four clades representing four orders (Figure 2). *Guinardia* and *Rhizosolenia* were recovered as the monophyletic clade Rhizosoleniales. In Rhizosoleniales, *R. setigera* was sister to *Guinardia* plus the left *Rhizosolenia* with high bootstrap support (100%), which shows that *R. setigera* may represent a hitherto undescribed genus (*pseudo-Rhizosolenia*) independent of *Guinardia* and *Rhizosolenia*. The controversial results that intraspecific genetic distance in *G. striata* (OM827251 and MG755796) exceeds interspecific genetic distance between *G. striata* and *G. delicatula* indicated that the *G. striata* strain (OM827251) we identified is not the same species as that (MG755796). More work needs to be carried out to reveal whether there are cryptic species in *Guinardia. Actinocyclus* and *Coscinodiscus* clustered together to form the clade Coscinodiacales which was sister to Rhizosoleniales. *Paralia* and *Stephanopyxis*, which represented two distinct orders, clustered tightly to form the Paraliales-Stephanopyxales complex. The Paraliales-Stephanopyxales complex was sister to Rhizosoleniales-Coscinodiscales complex with 100% bootstrap support (Figure 2).

**FIGURE 3:**
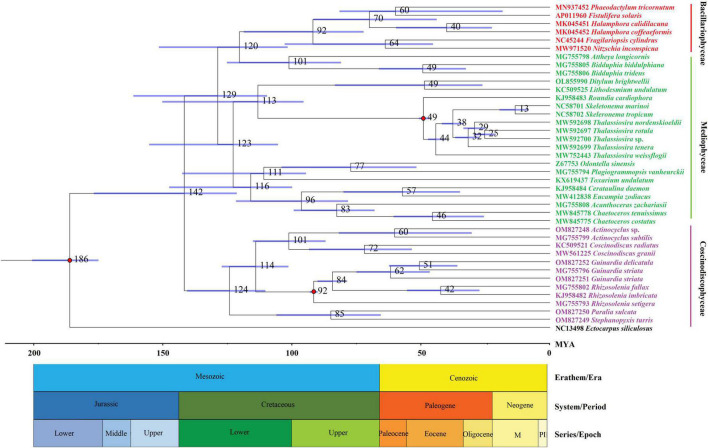
Time-calibrated phylogenetic analysis based on nucleotide sequences of 109 PCGs shared by the plastomes of selected diatom species as well as *Ectocarpus siliculosus* as outgroup. The fossil calibration taxa were indicated by red dots at corresponding nodes. Horizontal bars in blue color represented the 95% highest posterior density (HPD) values of the estimated divergence time.

A molecular dating tree was constructed to evaluate the divergence time in diatom lineages based on nt sequences of 109 PCGs shared by the plastomes of selected diatoms and *E. siliculosus*. Our results showed that ancient diatoms (Bacillariophyta) were estimated to appear at about 186 MYA, which was within the range of previous reports (Sorhannus, 2007; Medlin, 2015), while the Coscinodiscophyceae was separated from the Bacillariophyceae-Mediophyceae complex at about 142 MYA in the early Lower Cretaceous (Figure 3). These coscinodiscophycean diatom species were well recovered as a monophyletic clade (Figure 3). In Coscinodiscophyceae, the Paraliales-Stephanopyxales complex split at 124 MYA, followed by Coscinodiacales and Rhizosoleniales that split 114 MYA (Figure 3). The divergence time between Paraliales and Stephanopyxales was estimated at 85 MYA in the middle Upper Cretaceous, which was consistent with the time reported previously (Sorhannus, 2007). These results emphasize that Paraliales and Stephanopyxales appeared later than Coscinodiacales and Rhizosoleniales. In Coscinodiacales, *Actinocyclus* and *Coscinodiscus* split at about 101 MYA. In Rhizosoleniales, *R. setigera* emerged first at about 92 MYA and then the split between *Guinardia* and other *Rhizosolenia* occurred at 84 MYA.

### Variations of gene repertoires in coscinodiscophycean plastomes

**FIGURE 4:**
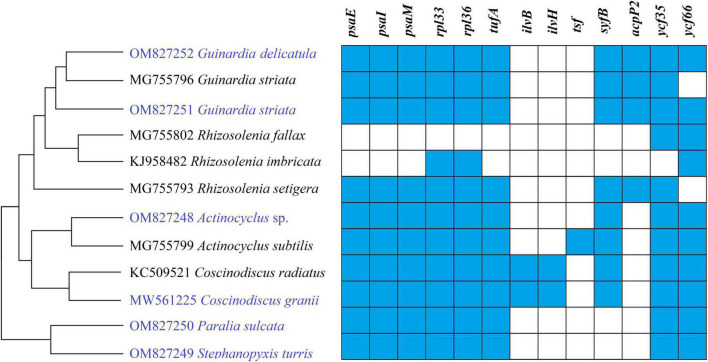
Changes in gene content of PCGs in the 12 coscinodiscophycean plastomes. The Maximum Likelihood (ML) tree on the left showed their phylogenetic relationships, and the matrix on the right showed the presence (blue) and absence (white) of 16 PCGs.

Gene repertoires in coscinodiscophycean plastomes showed substantial variations, ranging from 154 genes in *R. fallax* to 163 genes in two *Coscinodiscus* plastomes (Table 1 and Supplementary Table 3). Comparative analysis revealed that 120 PCGs, three ribosomal RNA genes (rRNAs), 27 transfer RNA genes (tRNAs) and two regulatory small RNA genes (sRNAs) were shared by these coscinodiscophycean plastomes, with 13 PCGs lost to various degrees in different lineages of Coscinodiscophyceae (Figure 4 and Table 2), which is most likely due to their horizontal transfer to corresponding nuclear genomes (Ruck et al., 2014; Liu et al., 2021a,b). Twenty-seven tRNAs were sufficient for messenger RNA translation in diatom plastomes which contained one more tRNA (*trnR3*) than the plastomes of brown algae (Liu et al., 2017). In addition to two copies of *trnN(guu)* located at IRa and IRb, respectively, another perfect copy of *trnN(guu)* which shared the same sequence as the formers was situated in SSC of the *Stephanopyxis* plastome, but was absent in other coscinodiscophycean plastomes, indicating that the duplication of *trnN(guu)* occurred only in the *Stephanopyxis* plastome. Two sRNAs, *ffs* and *ssrA*, were conservatively located in the adjacent upstream (5′) region of *psbX* and adjacent downstream (3′) region of *trnR3(ccg)*, respectively. *ffs* is a signal-recognition particle RNA that participates in the transmembrane transport of nuclear-encoded genes with plastid localization, and *ssrA* is a small regulatory RNA that interacts with stalled ribosomes to resume translation (Galachyants et al., 2012).

**TABLE 2:** Genes identified in these 12 diatom plastomes in Coscinodiscophyceae.

Functional classification	Genes*
Protein-coding genes (PCGs: 133)**	
Transcription and translation (52)	*rpl1*, *rpl2*, *rpl3*, *rpl4*, *rpl5*, *rpl6*, *rpl11*, *rpl12*, *rpl13*, *rpl14*, *rpl16*, *rpl18*, *rpl19*, *rpl20*, *rpl21*, *rpl22*, *rpl23*, *rpl24*, *rpl27*, *rpl29*, *rpl31*, *rpl32*, *rpl33*, *rpl34*, *rpl35*, *rpl36*, *rps2*, *rps3*, *rps4*, *rps5*, *rps6*, *rps7*, *rps8*, *rps9*, *rps10*, *rps11*, *rps12*, *rps13*, *rps14*, *rps16*, *rps17*, *rps18*, *rps19*, *rps20*, *dnaB*, *rpoA*, *rpoB*, *rpoC1*, *rpoC2*, *syfB*, *tsf*, *tufA*
Photosystem I (12)	*psaA*, *psaB*, *psaC*, *psaD*, *psaE*, *psaF*, *psaI*, *psaJ*, *psaL*, *psaM*, *ycf3*, *ycf4*
Photosystem II (19)	*psbA*, *psbB*, *psbC*, *psbD*, *psbE*, *psbF*, *psbH*, *psbI*, *psbJ*, *psbK*, *psbL*, *psbN*, *psbT*, *psbV*, *psbW*, *psbX*, *psbY*, *psbZ*, *ycf12*
Electron transport and ATP synthesis (18)	*atpA*, *atpB*, *atpD*, *atpE*, *atpF*, *atpG*, *atpH*, *atpI*, *ccs1*, *ccsA*, *petA*, *petB*, *petD*, *petF*, *petG*, *petL*, *petM*, *petN*
Carbon assimilation and metabolism (8)	*acpP1*, *acpP2*, *ilvB*, *ilvH*, *rbcL*, *rbcS*, *thiG*, *thiS*
Light harvesting and chl biosynthesis (1)	*chlI*
Signal transduction (2)	*cbbX*, *rbcR*
Protein import (4)	*secA*, *secG*, *secY*, *tatC*
Fe-S assembly (2)	*sufB*, *sufC*
Chaperones (2)	*dnaK*, *groEL*
Antioxidase and proteolysis (3)	*bas1*, *clpC*, *ftsH*
Conserved chloroplast PCGs (10)	*ycf33*, *ycf35*, *ycf39*, *ycf41*, *ycf45*, *ycf46*, *ycf66*, *ycf88*, *ycf89*, *ycf90*
Ribosomal RNA genes (rRNAs: 3)	*rnl*, *rns*, *rrn5*
Transfer RNA genes (tRNAs: 27)	*trnA(ugc)*, *trnC(gca)*, *trnD(guc)*, *trnE(uuc)*, *trnF(gaa)*, *trnG1(gcc)*, *trnG2(ucc)*, *trnH(gug)*, *trnI1(gau)*, *trnK(uuu)*, *trnL1(uaa)*, *trnL2(uag)*, *trnM1(cau)*, *trnM2(cau)*, *trnM3(cau)*, *trnN(guu)*, *trnP(ugg)*, *trnQ(uug)*, *trnR1(acg)*, *trnR2(ucu)*, *trnR3(ccg)*, *trnS1(gcu)*, *trnS2(uga)*, *trnT(ugu)*, *trnV(uac)*, *trnW(cca)*, *trnY(gua)*
Small RNA genes (sRNAs: 2)	*ffs*, *ssrA*

*Genes are classified according to their function. The underlined genes represent that these genes have experienced varying degrees of loss in different lineages of Coscinodiscophyceae.

**Numbers within parentheses indicate the number of genes in a specific functional group.

All these coscinodiscophycean cpDNAs belong to intron-less plastomes. The vast majority of sequenced diatom plastomes contain no intron, and only several diatom plastomes harbor a small number of introns. So far, plastid introns were detected in six housekeeping genes including *atpB*, *groEL*, *petB*, *petD*, *psaA*, and *rnl* among sequenced diatom plastomes (Brembu et al., 2014; Ruck et al., 2017; Yu et al., 2018; Hamsher et al., 2019; Gastineau et al., 2021; Górecka et al., 2021; Liu et al., 2021a). Overall, diatom plastomes exhibit an essential characteristic of being uncontaminated by intron. This characteristic is relatively similar in chloroplasts originating from secondary endosymbiosis (e.g., Phaeophyceae) (Liu et al., 2017), but significantly different from chloroplasts originating from primary endosymbiosis (e.g., Ulvophyceae and land plants), which typically contain more group I or/and group II introns in housekeeping genes (Liu et al., 2023).

Frequent loss of housekeeping PCGs were observed in these coscinodiscophycean plastomes, suggesting that diatom plastomes showed an ongoing reduction in gene content during evolution. Five PCGs including *ilvB*, *ilvH*, *tsf*, *syfB*, and *acpP2* were absent from plastomes of both *Paralia* and *Stephanopyxis*. Considering their phylogenetic relationship in Coscinodiscophyceae (Figure 4), it is most likely that the loss of these five PCGs happened after the split of the Paraliales-Stephanopyxales complex from the Rhizosoleniales-Coscinodiscales complex. Three PCGs including *ilvB*, *ilvH*, and *tsf* were missing in all of six Rhizosoleniales plastomes, suggesting that the loss of these three genes might occur earlier than the loss of other 10 PCGs in Rhizosoleniales. The plastomes of *Rhizosolenia fallax*-*imbricata* lost more PCGs compared with the other lineages (Yu et al., 2018). Especially, some housekeeping PCGs associated with photosystem I (*psaE*, *psaI*, and *psaM*) and ribosome (*rpl33* and *rpl36*) were lost. Combined with phylogenetic analysis, it can be seen that the loss of these genes in the *R. fallax*-*imbricata* plastomes should be recent events. Considering the important function of these genes, they are most likely transferred into the nuclear genome through endosymbiotic gene transfer. Similar events have been identified in other diatoms. For example, endosymbiotic gene transfers of *petF* into nuclear genomes were identified to occur in the plastomes of *Thalassiosira* and *Skeletonema* species (Liu et al., 2021a,b). The transfer of *petF* from plastome to nuclear genome is linked to ecological success of *Thalassiosira oceanica* (Lommer et al., 2010). In Coscinodiscophyceae, two PCGs, *ilvB* and *ilvH*, were only present in *Coscinodiscus* plastomes, while *tsf* were found only in *A. subtilis* plastome. These three PCGs have been lost multiple times in the evolution of diatoms, and nowadays are rarely scattered in plastomes of specific species in different lineages of diatoms (Yu et al., 2018). It was found that *tsf* was present in nuclear genomes of some diatoms (e.g., *Phaeodactylum* and *Thalassiosira* species) with *tsf*-lacking plastomes (Ruck et al., 2014).

**FIGURE 5:**
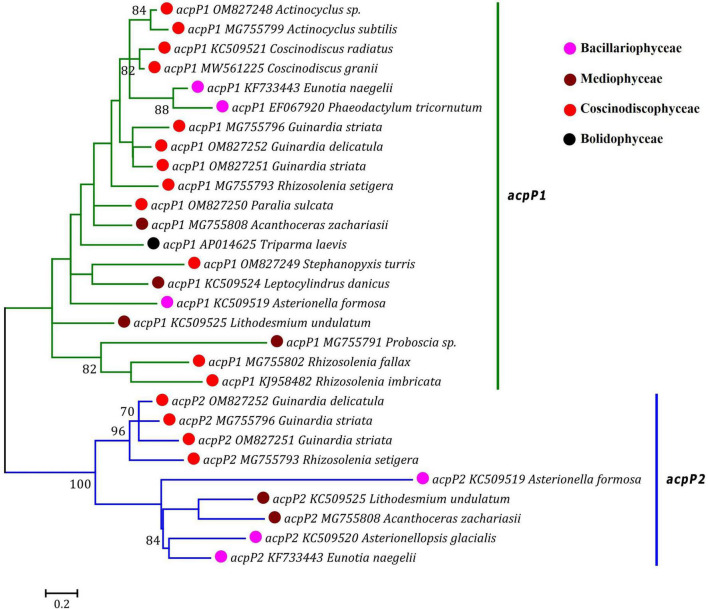
Unrooted Maximum Likelihood (ML) phylogenetic tree based on the amino acid sequences of AcpP1 and AcpP2 found in plastomes of diatoms and *Triparma laevis*. Numbers at the branches represented bootstrap values. The bootstrap support values greater than 70% were displayed at branches. Branch lengths were proportional to the amount of sequence change, which were indicated by the scale bar below the trees.

Based on the distribution pattern of two *acpP* genes (*acpP1* and *acpP2*) in diatom plastomes, *acpP* was previously considered to have experienced multiple independent duplications in the *acpP1/2*-containning plastomes, meanwhile it was believed to have undergone multiple losses in the *acpP*-lacking plastomes during the evolution of diatoms (Yu et al., 2018). Our phylogenetic analysis based on the AA sequences of AcpP1 and AcpP2 in the plastomes of diatoms and *T. laevis* revealed that the *acpP* genes were clearly grouped into two clades representing *acpP1* and *acpP2* lineages, respectively (Figure 5). Each clade harbored related members from Bacillariophyceae, Mediophyceae and Coscinodiscophyceae. These results indicated that *acpP1* and *acpP2* in diatom plastomes were not derived from multiple gene duplications occurring in different lineages of diatoms, instead they originated from an early gene duplication event occurred in the common progenitor after diatom emergence, followed by frequent loss of one or both genes (*acpP1* or/and *acpP2*) in different diatom lineages (Ruck et al., 2014).

### Variations of IRs and gene order in coscinodiscophycean plastomes

**FIGURE 6:**
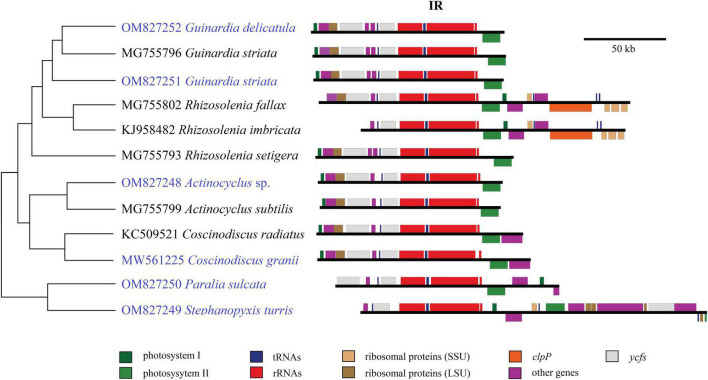
Comparison of inverted repeats (IRs) in the 12 coscinodiscophycean plastomes. The Maximum Likelihood (ML) tree on the left showed their phylogenetic relationships, and the photo on the right showed the structure and size of IRs. Annotated genes were colored according to their functional categories.

Size changes in these coscinodiscophycean plastomes were mainly caused by variations of IRs, which ranged from 11.3 kb in *A. curvatulus* to 20.1 kb in *S. turris*. Similar to most of the IR-containing plastomes, diatom IRs were composed of the conserved *rns*-*trnI*-*trnA*-*rnl*-*rrn5* gene block and additional genes that flanked the ribosomal gene operon. However, IRs of these coscinodiscophycean plastomes shared a much larger conserved gene block containing nine genes, i.e., *acpP*-*trnP*-*ycf89*- *rns*-*trnI*-*trnA*-*rnl*-*rrn5*-*psbA*. At the intrageneric level of *Guinardia*, *Actinocyclus* and *Coscinodiscus*, the structure and size of IRs show little fluctuations (Figure 6), suggesting that IRs were generally conserved among closely related species. However, IRs in *S. turris* and *R. fallax*-*imbricata* exhibited a similar trend of large expansion to SSCs and slightly small contraction from LSCs, which eventually led to the conspicuous increase in IR sizes. The most obvious expansion of IRs was observed in *Stephanopyxis* plastome with the 20.1-kb IR which harbored 23 genes and was much longer than those in other coscinodiscophycean lineages (Figure 6).

In the evolution of diatoms, the contraction and expansion of IRs occurred frequently in many lineages, which resulted in a huge variations of IR sizes, ranging from 6.8 kb in *Synedra acus* to 79.0 kb in *Climaconeis cf. scalaris*, an eleven-fold difference (Galachyants et al., 2012; Yu et al., 2018; Gastineau et al., 2021). Interestingly, it was found that the circular *Pseudo-nitzschia multiseries* plastome (KR709240) could lack IR (Cao et al., 2016; He et al., 2022), which may represent a new trend in the evolution of diatom plastomes if the assembly was correct. As found in the green algae (e.g., *Ulva*), IR is not an essential feature of plastomes (Liu and Melton, 2021; Liu et al., 2023), and it would change considerably in different eukaryotic photosynthetic lineages.

**FIGURE 7:**
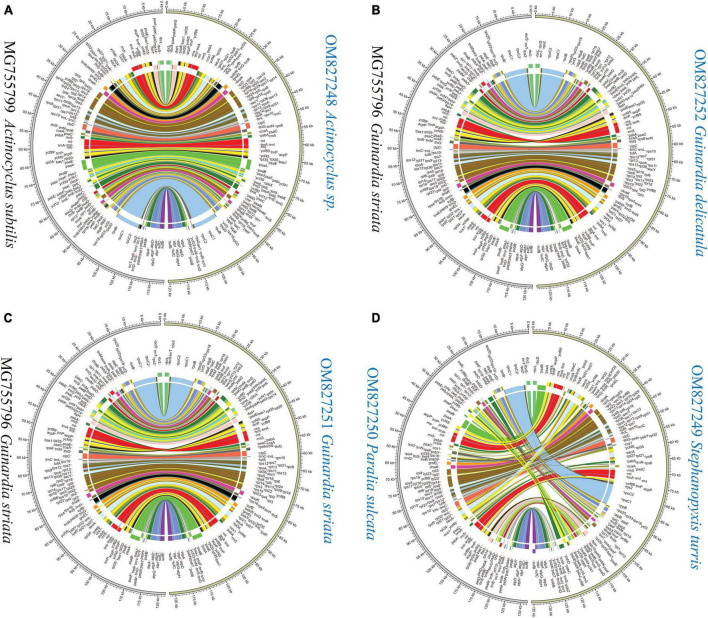
Pair-wise comparison of gene order and genome rearrangement between coscinodiscophycean plastomes. **(A)** Comparison of *Actinocyclus* sp. (OM827248) and *Actinocyclus subtilis* (MG755799). **(B)** Comparison of *Guinardia delicatula* (OM827252) and *Guinardia striata* (MG755796). **(C)** Comparison of *Guinardia striata* (OM827251) and *Guinardia striata* (MG755796). **(D)** Comparison of *Stephanopyxis turris* (OM827249) and *Paralia sulcate* (OM827250).

Mauve alignments showed that *Actinocyclus* and *Coscinodiscus* plastomes shared the same gene order and showed high collinearity at the intra-order level of Coscinodiacales (Supplementary Figure 1). At the intrageneric level of *Rhizosolenia*, multiple inversion and translocation events were identified (Supplementary Figure 1). Within the genus *Guinardia*, we observed that one of IR gene clusters composing of 14 genes was inverted in the *G. striata* plastome (MG755796) (Yu et al., 2018), which resulted in two IR gene clusters being arranged in a forward direction instead of a reverse direction (Figure 7 and Supplementary Figure 2). If this was not due to assembly issues, the emergence of this unusual IR rearrangement represents new evolutionary features that are significantly different from our newly sequenced *Guinardia* plastomes (OM827251 and OM827252) which maintain a usual arrangement of IRs.

Different diatom lineages showed significantly different evolution rates in gene order. Coscinodiacales and Rhizosoleniales appeared earlier than Paraliales and Stephanopyxales in the evolution of diatoms (Figure 3). Gene order in Coscinodiacales were highly conserved at the intra-order level, while multiple rearrangements were observed in Rhizosoleniales and between Paraliales and Stephanopyxales (Figure 7 and Supplementary Figure 1). Considering all instances of IR variations, it seems that variations of gene orders matched well with variations of IRs in these diatom plastomes, consistent with previous understanding that. IR regions might play an important role in stabilizing the architecture of plastomes (Turmel and Lemieux, 2018). It is reasonable to assume that accelerating IR changes involves frequent genome recombination and rearrangement (Figure 7 and Supplementary Figure 1), when considering substantial variation of the additional genes that flanked the ribosomal gene operon (*rns*-*trnI*-*trnA*-*rnl*-*rrn5*). However, despite frequent plastome rearrangements in these diatoms, the affiliation of gene clusters has not changed, suggesting that rearrangements were strictly restricted within LSC and SSC. This is different from what was observed in the IR-losing plastomes in green algae where gene clusters have undergone a larger range of frequent changes (Liu and Melton, 2021; Liu et al., 2023).

## Conclusion

Construction of six plastomes of diatom species in the class Coscinodiscophyceae in our work substantially boosted the number of assembled plastomes in this class, which in turn helped to reveal new evolutionary trends and details of diatom plastomes. The coscinodiscophycean plastomes in different lineages exhibited different evolutionary trends. The plastomes in Coscinodiacales were highly conserved in genome size, gene content, IR structure and gene order at the intra-order level, but plastomes in Rhizosoleniales and Paraliales-Stephanopyxales complex displayed multiple changes in the above aspects, even at the intrageneric level (e.g., *Rhizosolenia*). Comparative analysis revealed that 120 protein-coding genes (PCGs), three ribosomal RNA genes (rRNAs), 27 transfer RNA genes (tRNAs) and two regulatory small RNA genes (sRNAs) were shared by these coscinodiscophycean plastomes, but the other 13 PCGs were lost to varying degrees in different coscinodiscophycean lineages. The lost housekeeping PCGs could have been transferred into the nuclear genome via endosymbiotic gene transfer. Frequent loss of housekeeping PCGs were observed in these coscinodiscophycean plastomes, suggesting that diatom plastomes showed an ongoing reduction in gene content during evolution. The IRs in *S. turris* and *R. fallax*-*imbricata* showed a trend of expansion to the SSC and contraction from the LSC, which eventually led to the conspicuous increase in IR size. Different lineages of diatoms show significantly different evolution rates in gene order. As more genomic data accumulate in future, we will be able to depict more comprehensive evolutionary pathways and reveal evolution mechanism of species diversity in diatoms.

## Data availability statement

The datasets presented in this study can be found in online repositories. The names of the repository/repositories and accession number(s) can be found in this article/Supplementary material.

## Author contributions

NC designed the study. YW, HH, and FL performed the experiments. FL and YW performed the analysis. FL wrote the manuscript with contributions from coauthors. All authors have read and approved the final version of the manuscript.

## Footnotes

1
https://github.com/BFL-lab/Mfannot

2
https://www.ncbi.nlm.nih.gov/orffinder/
