# Supplementary material for: Evolutionary dynamics of plastomes in coscinodiscophycean diatoms revealed by comparative genomics
Source: Front Microbiol. 2023 Jun 15;14:1203780. doi: 10.3389/fmicb.2023.1203780 (PMC10307964; doi:10.3389/fmicb.2023.1203780)
Supplement: Supplementary file 1 [file Table_1.pdf]

Table S1. The 18S rDNA sequences of coscinodiscophycean diatoms used in this study.

**Species**	**Accession number (18S rDNA)**	**References**
Actinocyclus actinochilus	AY485506	GenBank
Actinocyclus curvatulus	X85401.2	GenBank
Actinocyclus sp.	MW750345	This study
Actinoptychus splendens	KJ577842	GenBank
Aulacodiscus orientalis	HQ912652	GenBank
Corethron hystrix	EF192981	GenBank
Coscinodiscus centralis	KP403782	GenBank
Coscinodiscus granii	MZ544480	This study
Coscinodiscus granii	HQ912667	GenBank
Coscinodiscus granii	AY485495	GenBank
Coscinodiscus jonesianus	KJ577852	GenBank
Coscinodiscus radiatus	X77705.2	GenBank
Endictya oceanica	KC309534	GenBank
Guinardia delicatula	MW750346	This study
Guinardia delicatula	AY485487	GenBank
Guinardia flaccida	AJ535191	GenBank
Guinardia striata	MW750344	This study
Guinardia striata	KT861015	GenBank
Hemidiscus cuneiformis	KY362440	GenBank
Hyalodiscus scoticus	AB430587	GenBank
Melosira dubia	AB430588	GenBank
Melosira varians	X85402.2	GenBank
Paralia longispina	KJ577865	GenBank
Paralia sulcata	MW750343	This study
Paralia sulcata	HQ912573	GenBank
Podosira stelligera	HQ912567	GenBank
Pseudosolenia calcar-avis	KJ577871	GenBank
Rhizosolenia imbricata	AJ535178	GenBank
Rhizosolenia similoides	AJ535177	GenBank
Stephanopyxis nipponica	M87330	GenBank
Stephanopyxis palmeriana	AY485527	GenBank
Stephanopyxis turris	MW750342	This study
Stephanopyxis turris	HQ912657	GenBank
Urosolenia eriensis	HQ912577	GenBank
