# Supplementary material for: Evolutionary dynamics of plastomes in coscinodiscophycean diatoms revealed by comparative genomics
Source: Front Microbiol. 2023 Jun 15;14:1203780. doi: 10.3389/fmicb.2023.1203780 (PMC10307964; doi:10.3389/fmicb.2023.1203780)
Supplement: Supplementary file 2 [file Table_2.pdf]

Table S2. Species identification of six diatom strains in the class Coscinodiscophycae based on similarity analysis of 18S rDNA sequences.

**Strains**	**Species**	**Accession number (18S rDNA)**	**Reference sequence with the highest similarity**	**Similarity (%)**
CNS00558	*Guinardia delicatula*	MW750346	AY485487	99.94
CNS00513	*Guinardia striata*	MW750344	KT861015	99.63
CNS00114	*Actinocyclus* sp.	MW750345	X85401	98.86
CNS00554	*Coscinodiscus granii*	MZ544480	HQ912667	99.94
CNS00378	*Stephanopyxis turris*	MW750342	HQ912657	99.63
CNS00428	*Paralia sulcata*	MW750343	HQ912573	99.71
