# Supplementary material for: Evolutionary dynamics of plastomes in coscinodiscophycean diatoms revealed by comparative genomics
Source: Front Microbiol. 2023 Jun 15;14:1203780. doi: 10.3389/fmicb.2023.1203780 (PMC10307964; doi:10.3389/fmicb.2023.1203780)
Supplement: Supplementary file 3 [file Table_3.pdf]

Table S3. Comparison of gene content of 12 diatom plastomes in Coscinodiscophyceae.

Species	Gene content	Gene content in IRs	Net gene content*
	**PCGs/rRNAs/tRNAs/sRNAs**	**PCGs/rRNAs/tRNAs**	**PCGs/rRNAs/tRNAs/sRNAs**
Guinardia delicatula	139/6/30/2	9/3/3	130/3/27/2
Guinardia striata	139/6/30/2	9/3/3	130/3/27/2
Guinardia striata	138/6/30/2	9/3/3	129/3/27/2
Rhizosolenia setigera	138/6/30/2	9/3/3	129/3/27/2
Rhizosolenia fallax	137/6/33/2	15/3/6	122/3/27/2
Rhizosolenia imbricata	134/6/33/2	11/3/6	123/3/27/2
Actinocyclus sp.	137/6/30/2	8/3/3	129/3/27/2
Actinocyclus subtilis	138/6/30/2	8/3/3	130/3/27/2
Coscinodiscus granii	140/6/30/2	9/3/3	131/3/27/2
Coscinodiscus radiatus	140/6/30/2	9/3/3	131/3/27/2
Paralia sulcata	135/6/30/2	7/3/3	128/3/27/2
Stephanopyxis turris	143/6/33/2	15/3/5	128/3/27/2

* Multicopy genes were counted once, e.g. the canonical genes located in IRs.
