# Supplementary material for: Evolutionary dynamics of plastomes in coscinodiscophycean diatoms revealed by comparative genomics
Source: Front Microbiol. 2023 Jun 15;14:1203780. doi: 10.3389/fmicb.2023.1203780 (PMC10307964; doi:10.3389/fmicb.2023.1203780)
Supplement: Supplementary file 4 [file Data_Sheet_1.PDF]

A

B

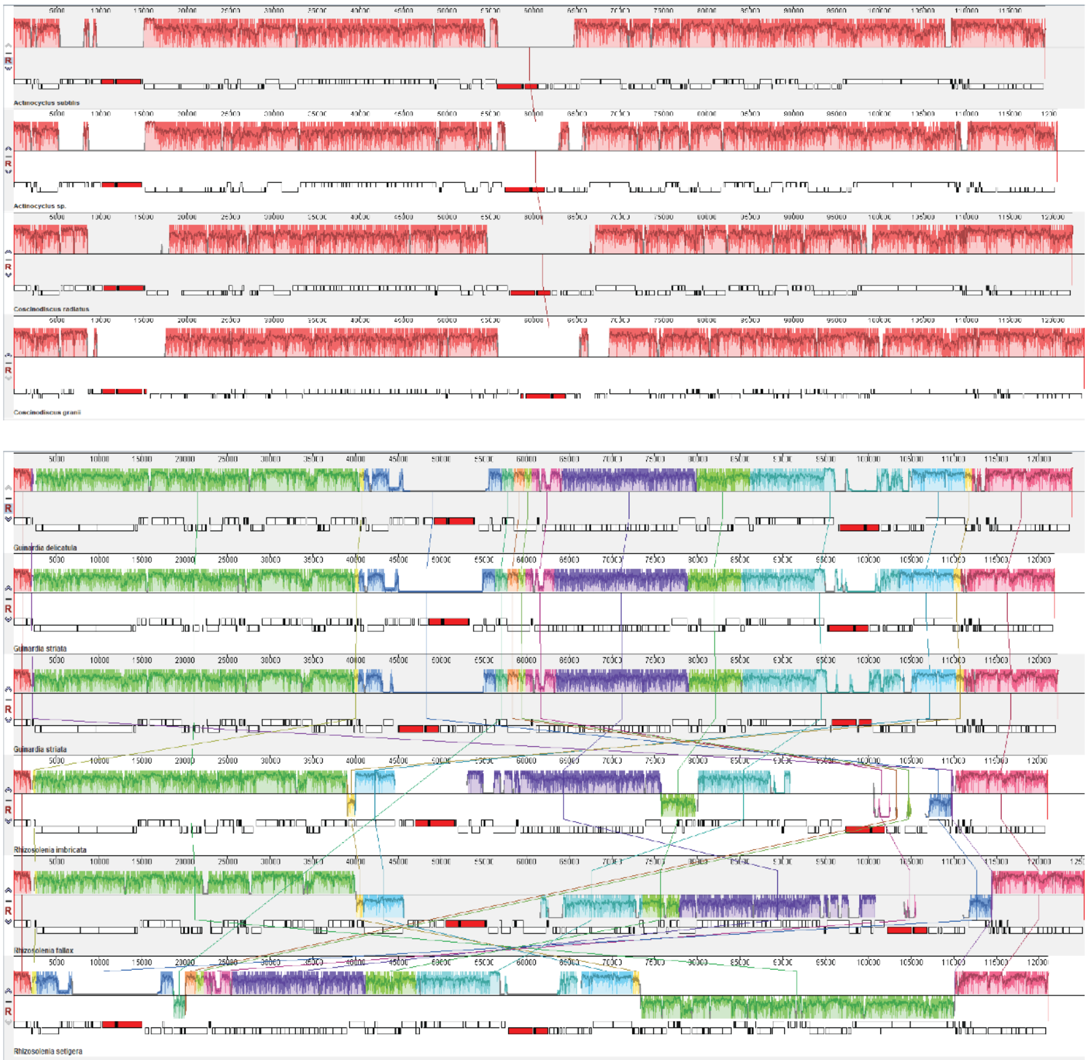

Fig. S1 Synteny comparison of Coscinodiscophyceae plastomes using the Mauve software. (A) Synteny analysis of four Coscinodiacales plastomes. (B) Synteny analysis of six Rhizosoleniales plastomes. Each colored block indicated a synteny block in these plastomes.
