# Supplementary material for: Evolutionary dynamics of plastomes in coscinodiscophycean diatoms revealed by comparative genomics
Source: Front Microbiol. 2023 Jun 15;14:1203780. doi: 10.3389/fmicb.2023.1203780 (PMC10307964; doi:10.3389/fmicb.2023.1203780)
Supplement: Supplementary file 5 [file Data_Sheet_2.PDF]

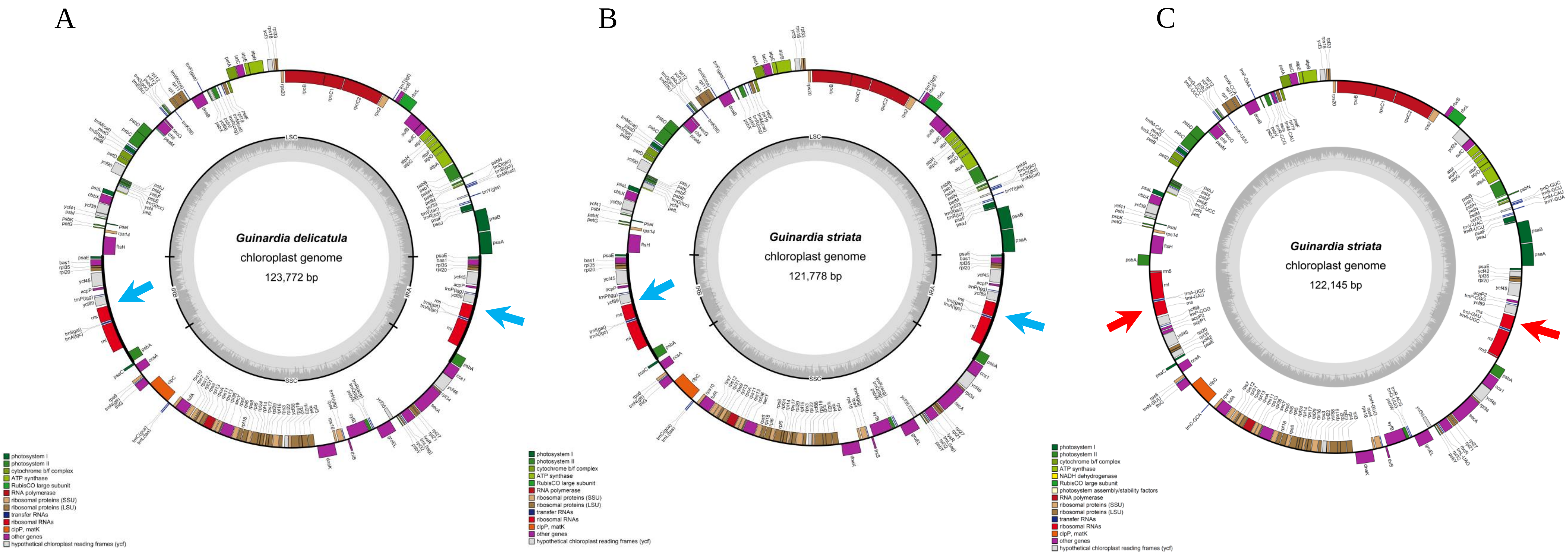

Fig. S2 Comparison of plastome structure in *Guinardia*. (A) The plastome in *Guinardia delicatula* (OM827252) with IRs (blue arrows). (B) The plastome in *Guinardia striata* (OM827251) with IRs (blue arrows). (C) The plastome in *Guinardia striata* (MG755796). One of IR gene clusters was inverted in the *G. striata* plastome, which resulted in two IR gene clusters being arranged in a forward direction instead of a reverse direction (red arrows).
